# Supplementary material for: Targeting NADPH Oxidase and Integrin α5β1 to Inhibit Neutrophil Extracellular Traps-Mediated Metastasis in Colorectal Cancer
Source: Int J Mol Sci. 2023 Nov 6;24(21):16001. doi: 10.3390/ijms242116001 (PMC10650826; doi:10.3390/ijms242116001)
Supplement: Supplementary file 1 [file ijms-24-16001-s001.zip › Supplementary materials_Final.pdf]

Targeting NADPH oxidase and integrin α5β1 to inhibit neutrophil extracellular traps-mediated metastasis in colorectal cancer

Wenyuan Zhu[1,2], Siqi Yang[1,2], Delan Meng[1,2], Qingsong Wang[1,2,*], Jianguo Ji[1,2,*]

[1] State Key Laboratory of Protein and Plant Gene Research, School of Life Sciences, Peking University, Beijing, 100871, China.

[2] Department of Biochemistry and Molecular Biology, School of Life Sciences, Peking University, Beijing, 100871, China.

Supplementary materials

1. Supplementary Tables. S1–S5.

2. Supplementary Figures. S1–S9.

Supplementary Tables

Supplementary Table S1. Pathological information of CRC patients for plasma NETs protein markers detection

Information		Num (%)	Information		Num (%)
Sex	Male	8 (40%)	Stage	Ⅰ	5 (25%)
	Female	12 (60%)		Ⅱ	5 (25%)
Age	> 60	14 (70%)		Ⅲ	5 (25%)
	< 60	6 (30%)		Ⅳ	5 (25%)
Site*	Right	5 (25%)	T	1	1 (5%)
	Left	15 (75%)		2	4 (20%)
Differentiation degree	High	1 (5%)		3	7 (35%)
	Medium/Low	19 (95%)		4	8 (40%)
Nerve infiltration	0	12 (60%)	N	0	11 (55%)
	1	8 (40%)		1	9 (45%)
Vascular infiltration	0	13 (65%)	M	0	14 (70%)
	1	7 (35%)		1	6 (30%)

Site*: Right: cecum, ascending colon, transverse colon, hepatic flexure of colon; Left: descending colon, rectosigmoid, rectum, sigmoid colon.

Supplementary Table S2. Pathological information of CRC patients for quantitative proteomic analysis of neutrophils

Information		Num (%)	Information		Num (%)
Sex	Male	20 (80%)	Stage	Ⅰ	5 (20%)
	Female	5 (20%)		Ⅱ	9 (36%)
Age	> 60	17 (68%)		Ⅲ	9 (36%)
	< 60	8 (32%)		Ⅳ	2 (8%)
Site*	Right	3 (12%)	T	1	2 (8%)
	Left	22 (88%)		2	4 (16%)
Differentiation degree	High	1 (4%)		3	10 (40%)
	Medium	24 (96%)		4	9 (36%)
Nerve infiltration	0	19 (76%)	N	0	16 (64%)
	1	6 (24%)		1	9 (36%)
Vascular infiltration	0	17 (92%)	M	0	23 (92%)
	1	8 (8%)		1	2 (8%)

Site*: Right: cecum, ascending colon, transverse colon, hepatic flexure of colon; Left: descending colon, rectosigmoid, rectum, sigmoid colon.

Supplementary Table S3. shRNA and PCR primers used in the present study

Primer	Sequences (5' to 3')
sh-NC-F	CCGGTTCTCCGAACGTGTCACGTTTCTCGAGAAACGTGACACGTTCGGA GAATTTTTCTAGAG
sh-NC-R	AATTCTCTAGAAAAAATTCTCCGAACGTGTCACGTTTCTCGAGAAACGT GACACGTTCGGAGAA
sh-SPARC-1	CCGGGCAGAGGTGACTGAGGTATTTCTCGAGAAATACCTCAGTCACCT CTGCTTTTTTCTAGAG
sh-SPARC-2	CCGGGCCACTTCTTTGCCACAAATTTCTCGAGAAATTTGTGGCAAAGAA GTGGCTTTTTTCTAGAG
sh-SPARC-3	CCGGGGACTTCGAGAAGAACTATTTCTCGAGAAATAGTTCTTCTCGAAG TCCTTTTTTCTAGAG
sh-ITGA5-1	CCGGCCACTGTGGATCATCATCCTACTCGAGTAGGATGATGATCCACAG TGGTTTTT
sh-ITGA5-2	CCGGCTCCTATATGTGACCAGAGTTCTCGAGAACTCTGGTCACATATAG GAGTTTTT
sh-ITGA5-3	CCGGCCACAGATAACTTCACCCGAACTCGAGTTCGGGTGAAGTTATCTG TGGTTTTT
sh-ITGB1-1	CCGGGCCTTGCATTACTGCTGATATCTCGAGATATCAGCAGTAATGCAA GGCTTTTT

sh-ITGB1-2	CCGGCCAAATCATGTGGAGAATGTACTCGAGTACATTCTCCACATGATT TGGTTTTT
sh-ITGB1-3	CCGGGCCCTCCAGATGACATAGAAACTCGAGTTTCTATGTCATCTGGAG GGCTTTTT
SPARC-3×Flag-F	TGCGGCCGCGAATTCATCGATATGAGGGCCTGGATCTTCTTTC
SPARC-3×Flag-R	GTCAGCCCGGGATCCTCTAGAGATCACAAGATCCTTGTCGATATCC

Supplementary Table S4. Real-time PCR primers used in the present study

Primer	Sequences (5' to 3')
GAPDH-F	ACAACTTTGGTATCGTGGAAGG
GAPDH-R	GCCATCACGCCACAGTTTC
PAD4-F	CAGGGGACATTGATCCGTGTG
PAD4-R	GGGAGGCGTTGATGCTGAA
SPARC-F	AGCACCCCATTGACGGGTA
SPARC-R	GGTCACAGGTCTCGAAAAAGC
Ki67-F	ACGCCTGGTTACTATCAAAAGG
Ki67-R	CAGACCCATTTACTTGTGTTGGA
PCNA-F	GCGTGAACCTCACCAGTATGT
PCNA-R	TCTTCGGCCCTTAGTGTAATGAT
c-Myc-F	GGCTCCTGGCAAAAGGTCA
c-Myc-R	CTGCGTAGTTGTGCTGATGT
MCM2-F	TGCCAGCATTGCTCCTTCCATC
MCM2-R	AAACTGCGACTTCGCTGTGCCA
E-cadherin-F	TACACTGCCCAGGAGCCAGA
E-cadherin-R	TGGCACCAGTGTCCGGATTA
N-cadherin-F	TTTGATGGAGGTCTCCTAACACC
N-cadherin-R	ACGTTTAACACGTTGGAAATGTG
Snail-F	CACTATGCCGCGCTCTTTC

Snail-R	GCTGGAAGGTAAACTCTGGATTAGA
Vimentin-F	TGAGTACCGGAGACAGGTGCAG
Vimentin-R	TAGCAGCTTCAACGGCAAAGTTC
Twist-F	AGTCCGCAGTCTTACGAGGA
Twist-R	GCCAGCTTGAGGGTCTGAAT
α-SMA-F	CGTGGCTACTCCTTCGTG
α-SMA-R	TGATGACCTGCCCGTCT
MMP2-F	TACAGGATCATTGGCTACACACC
MMP2-R	GGTCACATCGCTCCAGACT
MMP9-F	TGTACCGCTATGGTTACACTCG
MMP9-R	GGCAGGGACAGTTGCTTCT
PI3K-F	CCACGACCATCATCAGGTGAA
PI3K-R	CCTCACGGAGGCATTCTAAAGT
AKT1-F	AGCGACGTGGCTATTGTGAAG
AKT1-R	GCCATCATTCTTGAGGAGGAAGT
AKT2-F	ACCACAGTCATCGAGAGGACC
AKT2-R	GGAGCCACACTTGTAGTCCA
NCF1-F	GGGGCGATCAATCCAGAGAAC
NCF1-R	GTACTCGGTAAGTGTGCCCTG
NCF2-F	CCCACTCCCGGATTTGCTTC
NCF2-R	GTCTCGGTTAATGCTTCTGGTAA

NCF4-F	CGGGCCGAGAGTGACTTTG
NCF4-R	TCTTCACCTCGATGACGAAAAC
CYBB-F	ACCGGGTTTATGATATTCCACCT
CYBB-R	GATTTCGACAGACTGGCAAGA
PRKCA-F	GTCCACAAGAGGTGCCATGAA
PRKCA-R	AAGGTGGGGCTTCCGTAAGT
PRKCB-F	AGCCCCACGTTTTGTGACC
PRKCB-R	GCTGGGAACATTCATCACGC
PRKCG-F	AGCCACAAGTTCACCGCTC
PRKCG-R	GGACACTCGAAGGTCACAAAT
CYBA-F	CCCAGTGGTACTTTGGTGCC
CYBA-R	GCGGTCATGTACTTCTGTCCC
MEK1-F	CAATGGCGGTGTGGTGTTC
MEK1-R	GATTGCGGGTTTGATCTCCAG
MEK2-F	CCAAGGTCGGCGAACTCAAA
MEK2-R	TCTCAAGGTGGATCAGCTTCC
ERK1-F	CTACACGCAGTTGCAGTACAT
ERK1-R	CAGCAGGATCTGGATCTCCC
ERK2-F	TACACCAACCTCTCGTACATCG
ERK2-R	CATGTCTGAAGCGCAGTAAGATT

Supplementary Table S5. Antibodies used in the present study

Antibody	WB	IF	IHC	Specificity	Source	Catalog number
β-Actin	1:10000	/	/	Rabbit	Proteintech	19446-1-AP
GAPDH	1:10000	/	/	Mouse	Proteintech	60004-1-Ig
α-Tubulin	1:10000	/	/	Rabbit	Beyotime	AF5012
MPO	1:5000	1:200	/	Mouse	Proteintech	66177-1-Ig
IL-6	1:2000	/	/	Mouse	Beyotime	AF0201
Histone H3 (citrulline R2 + R8 + R17)	1:500	1:200	1:200	Rabbit	Abcam	ab5103
SPARC	1:750	1:200	/	Rabbit	Abcam	ab207743
Flag-tag	1:5000	/	/	Rabbit	CST	14793S
ITGB1	1:5000	1:500	/	Rabbit	Abcam	ab52971
ITGA5	1:5000	1:500	/	Rabbit	Abcam	ab150361
FAK	1:3000	/	/	Rabbit	Abcam	ab40794
Phospho-FAK (pY397)	1:1000	/	/	Rabbit	Abcam	ab81298
SRC	1:3000	/	/	Rabbit	Abcam	ab109381
Phospho-SRC (pY418)	1:500	/	/	Rabbit	Abcam	ab40660
RHOA	1:5000	/	/	Rabbit	Abcam	ab187027
ROCK1 + ROCK2	1:5000	/	/	Rabbit	Abcam	ab45171
Akt 1/2/3	1:5000	/	/	Rabbit	Beyotime	AF1789
Phospho-Akt (pSer473)	1:1000	/	/	Rabbit	CST	4060T
Ki67	1:2000	/	1:200	Rabbit	Abcam	ab92742
PKC	1:2000	/	/	Rabbit	Abcam	ab179522

Phospho-PKC (pT514)	1:1000	/	/	Rabbit	Abcam	ab109539
CYBA (p22 phox)	1:500	/	/	Mouse	Santa Cruz	sc-271968
CYBB (gp91 phox)	1:3000	/	/	Rabbit	Abcam	ab129068
Rac1+2+3	1:3000	/	/	Rabbit	CST	2465T
NCF1 (p47 phox)	1:3000	/	/	Rabbit	Abcam	ab181090
Phospho-NCF1 (pS370)	1:1000	/	/	Rabbit	Bioworld	BS4852
NCF2 (p67 phox)	1:3000	/	/	Rabbit	Abcam	ab109366
NCF4 (p40 phox)	1:3000	/	/	Rabbit	Abcam	ab76158
Phospho-NCF4 (pT154)	1:1000	/	/	Rabbit	Abclonal	AP1193
ERK1/2	1:3000	/	/	Mouse	Santa Cruz	sc-514302
Phospho-ERK1/2 (pT202/pY204)	1:1000	/	/	Mouse	Santa Cruz	sc-81492
MEK1/2	1:3000	/	/	Rabbit	CST	8727T
Phospho-MEK1/2 (pSer217/221)	1:1000	/	/	Rabbit	CST	9154T
RACK1	1:5000	1:200	/	Rabbit	Abcam	ab62735
Vimentin	1:5000	/	/	Rabbit	CST	5741T
Snail	1:2000	/	/	Rabbit	CST	3879T
CD4	/	1:200	/	Rabbit	Servicebio	GB11064
CD8	/	1:500	/	Rabbit	Servicebio	GB114196
Ly6G	/	/	1:200	Rabbit	Servicebio	GB11229
Goat anti-rabbit IgG-secondary antibody, Alexa Fluor 488	/	1:1000	/	Goat	Invitrogen	A11034

Goat anti-rabbit IgG-secondary antibody, Alexa Fluor 568	/	1:1000	/	Goat	Invitrogen	A11036
Goat anti-rabbit IgG-secondary antibody, Alexa Fluor 647	/	1:1000	/	Goat	Invitrogen	A21245

Supplementary Figure S1

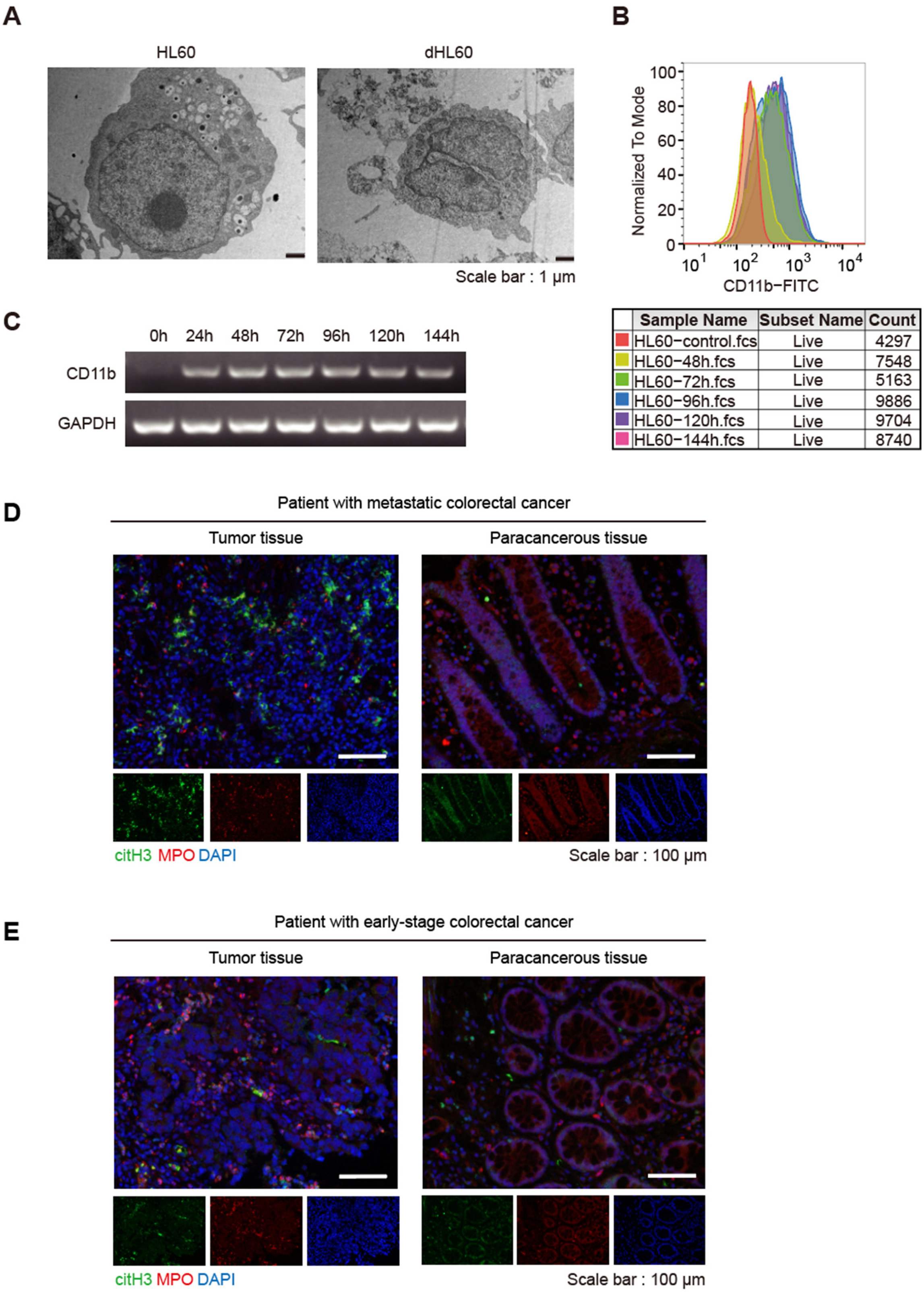

Supplementary Figure S1. Construction of the neutrophil cell model and immunofluorescence observation of NETs. (A) Transmission electron microscopy images of HL60 cells and differentiated HL60 cells induced by 1.25% DMSO. HL60 cells have round nuclei and differentiated HL60 nuclei display a multi-lobed karyotype. Scale bar, 1 μm. (B) The expression levels of CD11b at different timepoints was detected by flow cytometry. Following the completion of differentiation, the expression of CD11b at the plasma membrane gradually increases. (C) PCR detection of the expression levels of CD11b at different timepoints. (D) Representative immunofluorescence images of NETs in tissues from patient with metastatic CRC. NETs were stained for MPO (red), citH3 (green), and nuclei (blue). Scale bar, 100 μm. (E) Representative immunofluorescence images of NETs in tissues from patient with early-stage CRC. NETs were stained for MPO (red), citH3 (green), and nuclei (blue). Scale bar, 100 μm. Non-significant (ns) $p > 0.05$.

Supplementary Figure S2

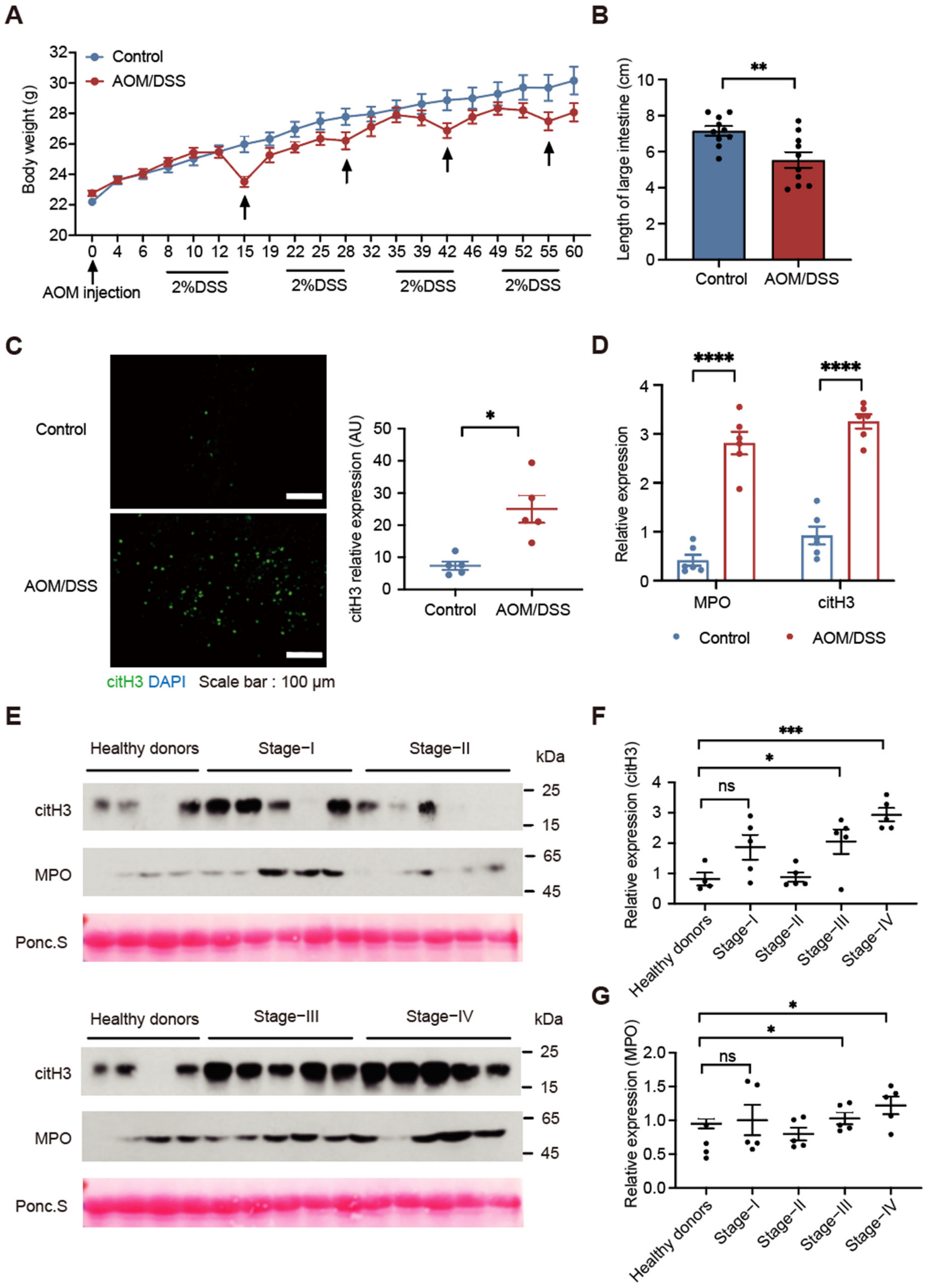

Supplementary Figure S2. Construction of the AOM/DSS-induced CRC murine model. (A) Bodyweight curve for AOM/DSS-induced CRC mice (n = 10 per group; mean ± SEM). (B) The length of the large intestine is significantly shorter in AOM/DSS-induced CRC mice (n = 10 per group; mean ± SEM, Student's t-test). (C) Representative images of citH3 immunofluorescence of intestines from AOM/DSS-induced CRC mice. Scale bar, 100 μm. Fluorescence intensity was analyzed using ImageJ (version 1.53r) (n = 5 per group; mean ± SEM, Student's t-test). (D) Quantification analysis of MPO and citH3 in plasma from AOM/DSS-induced CRC mice and control mice with Ponceau S (Ponc.S) as an internal control. Relative expression was analyzed using ImageJ (version 1.53r) (n = 6 per group; mean ± SEM, Student's t-test). (E) Western blotting analysis of citH3 and MPO expression in plasma from CRC patients and healthy donors. (F) Quantification analysis of citH3 in plasma from CRC patients and healthy donors with Ponceau S (Ponc.S) as an internal control. Relative expression was analyzed using ImageJ (version 1.53r) (Healthy donors: n = 4, CRC patients: n = 5 per group; mean ± SEM, Student's t-test). (G) Quantification analysis of MPO in plasma from CRC patients and healthy donors with Ponceau S (Ponc.S) as an internal control. Relative expression was analyzed using ImageJ (version 1.53r) (Healthy donors: n = 4, CRC patients: n = 5 per group; mean ± SEM, Student's t-test). Non-significant (ns) $p > 0.05$, * $p < 0.05$, ** $p < 0.01$, *** $p < 0.001$, **** $p < 0.0001$.

Supplementary Figure S3

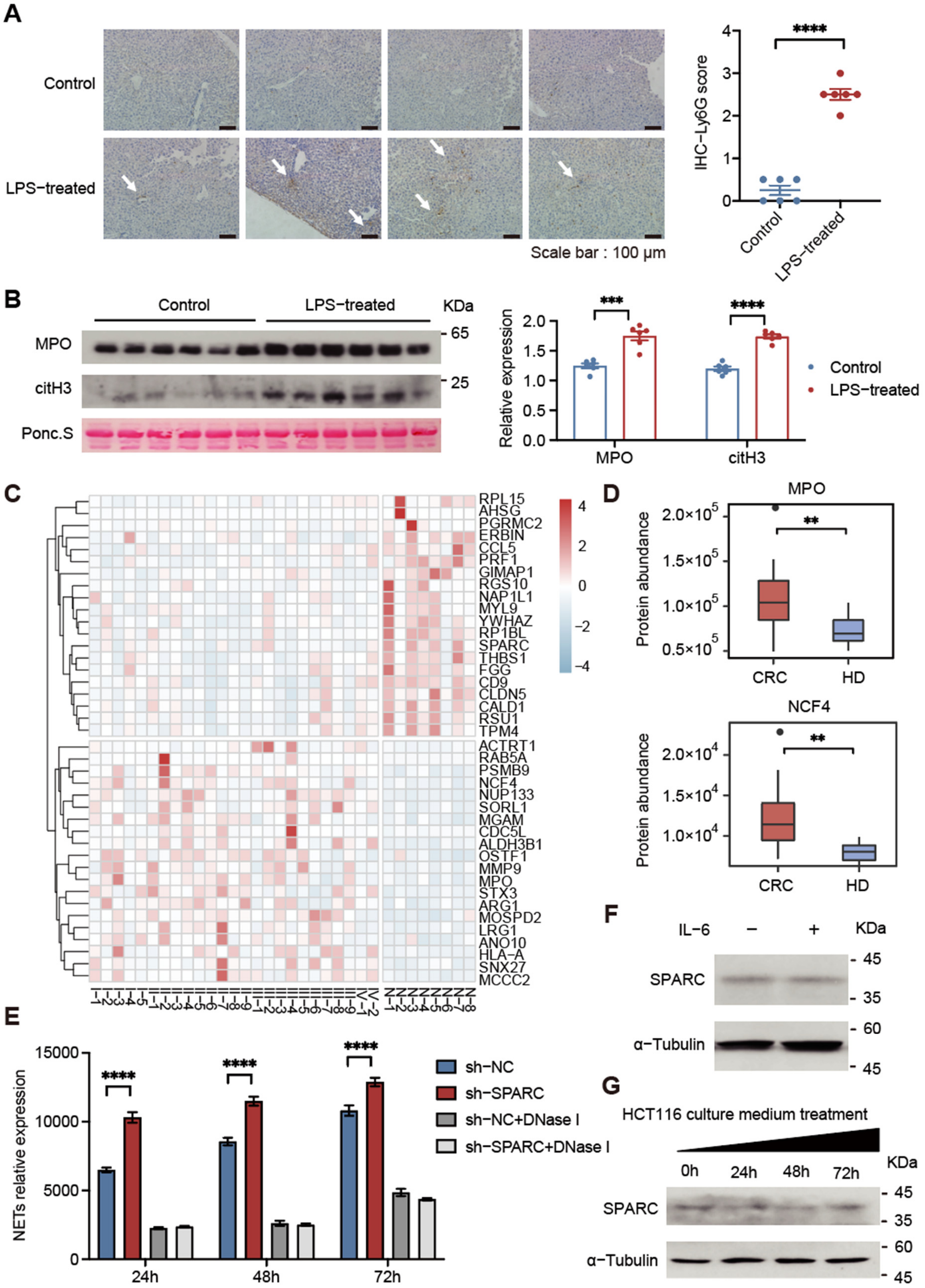

Supplementary Figure S3. Characterization of LPS-treated mice and the CRC neutrophil proteome. (A) Representative images of Ly6G immunohistochemistry staining of the liver from LPS-induced NETs mice. White arrows indicate typical neutrophil infiltration areas (Left). Scale bar, 100 µm. Immunohistochemical scoring statistics (Right) (n = 6 per group; mean ± SEM, Student's *t*-test). (B) Western blotting analysis of citH3 and MPO expression in plasma from LPS-treated mice and control (Left). Quantification analysis of MPO and citH3 in plasma from control mice and LPS-treated mice with Ponceau S (Ponc.S) as an internal control (Right). Relative expression was analyzed using ImageJ (version 1.53r) (n = 6 per group; mean ± SEM, Student's *t*-test). (C) Heatmap of significantly changed proteins in neutrophils from CRC patients. Colors represent the expression level: red indicates high expression and blue indicates low expression. (D) The abundance of MPO and neutrophil cytosol factor 4 (NCF4) in neutrophils from healthy donors and CRC patients. MPO and NCF4 are significantly upregulated (adjusted *p*-value was calculated by DESeq2, Wald-test). (E) Fluorescence quantitation of NETs DNA released in sh-SPARC and sh-NC dHL60 cells at different timepoints. DNase I was added 30 min prior to quantitation (n = 6 per group; mean ± SEM, Student's *t*-test). (F) Western blotting analysis of SPARC expression in HL60 cells with or without interleukin-6 (IL-6) stimulation. (G) Western blotting analysis of SPARC expression in HL60 cells with HCT116 cells culture medium treatment at different timepoints. Non-significant (ns) $p > 0.05$, ** $p < 0.01$, *** $p < 0.001$, **** $p < 0.0001$.

Supplementary Figure S4

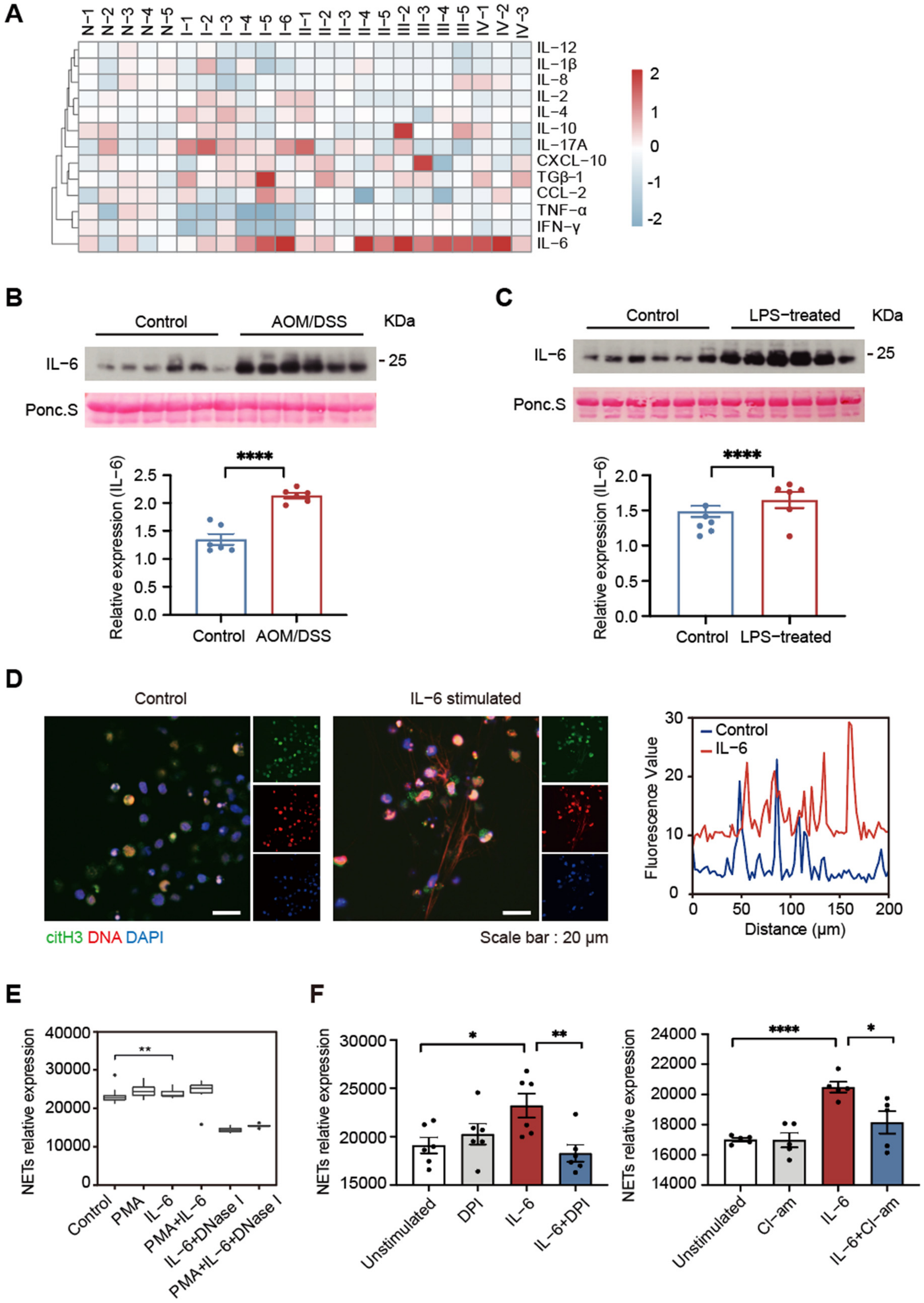

Supplementary Figure S4. IL-6 promotes NETs formation. (A) Heatmap of the expression levels of 13 cytokines in plasma from CRC patients (n = 18) and healthy donors (n = 5). Colors represent the expression levels: red indicates high expression and blue indicates low expression. (B) Western blotting analysis of IL-6 expression in plasma from AOM/DSS induced CRC mice and control mice (Up). Quantification analysis of IL-6 with Ponceau S (Ponc.S) as an internal control (Down). Relative expression was analyzed using ImageJ (version 1.53r) (n = 6 per group; mean ± SEM, Student's t-test). (C) Western blotting analysis of IL-6 expression in plasma from LPS-treated mice and control mice (Up). Quantification analysis of IL-6 with Ponceau S (Ponc.S) as an internal control (Down). Relative expression was analyzed using ImageJ (version 1.53r) (n = 6 per group; mean ± SEM, Student's t-test). (D) Representative immunofluorescence images of NETs formation in dHL60 cells with or without IL-6 stimulation. Cells were stimulated for 4 h and NETs were stained for extracellular DNA (red), nuclei (blue), and citH3 (green). Scale bar, 20 μm. The fluorescence intensity (n = 4 per group) was analyzed using ImageJ (version 1.53r). (E) Fluorescence quantitation of NETs DNA released by dHL60 cells after stimulation with IL-6 or PMA for 4 h. DNase I was added 30 min prior to quantitation (n = 8 per group; n = 5 in DNase I groups; mean ± SEM, Student's t-test). (F) Fluorescence quantitation of NETs DNA released by IL-6-stimulated dHL60 cells after treatment with the NADPH pathway inhibitor diphenyleneiodonium chloride (DPI) (20 μM) or the PAD4 inhibitor Cl-amidine (Cl-am) (10 μM) (DPI, n = 6; Cl-am, n = 5; mean ± SEM, Student's t-test). Non-significant (ns) $p > 0.05$, * $p < 0.05$, ** $p < 0.01$, **** $p < 0.0001$.

Supplementary Figure S5

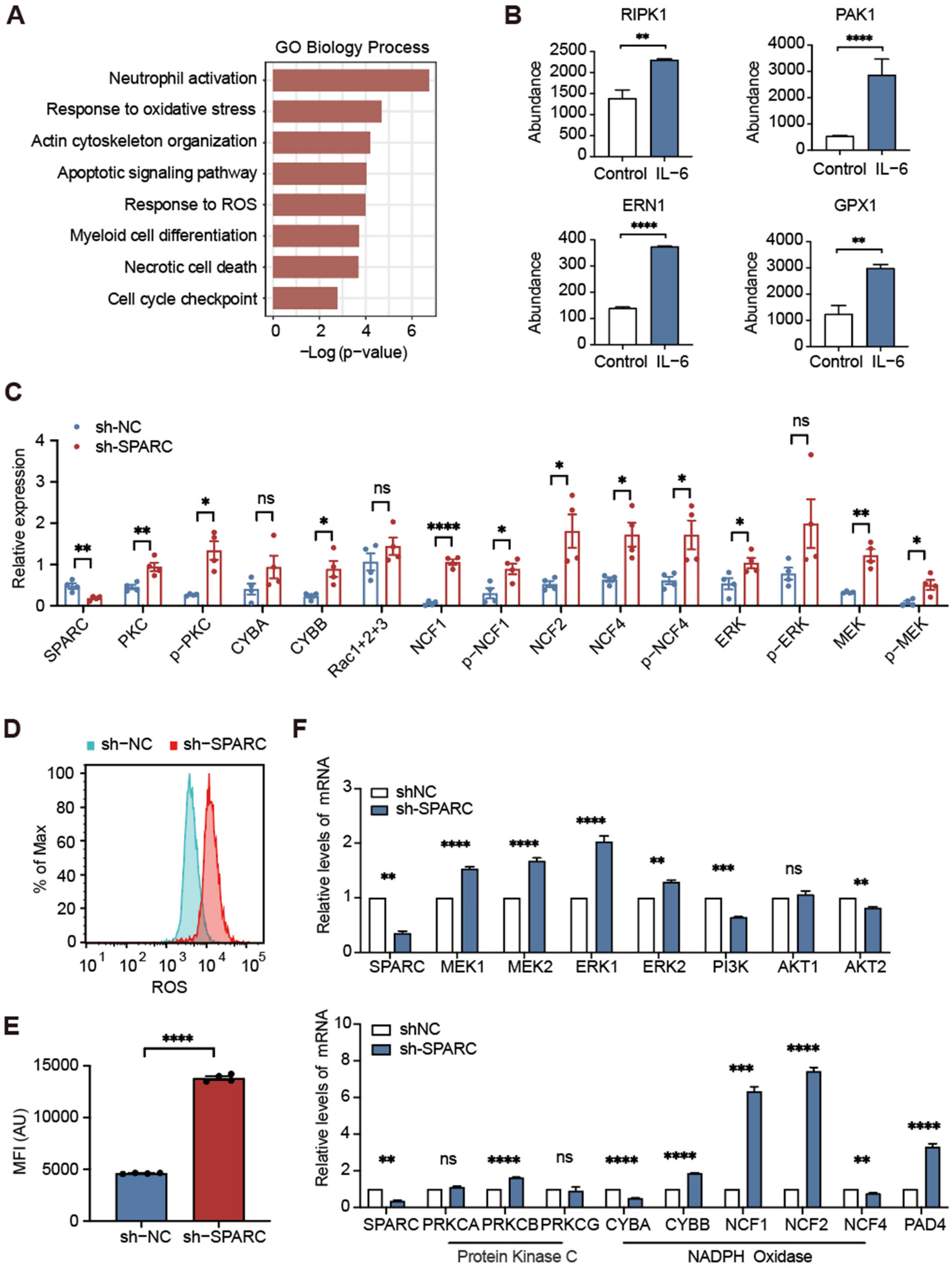

Supplementary Figure S5. SPARC affects NADPH oxidase-related pathways. (A) Biological function annotation enrichment analysis of significantly upregulated proteins in IL-6-stimulated HL60 cells. (B) Change in abundance of receptor-interacting serine/threonine-protein kinase 1 (RIPK1), serine/threonine-protein kinase PAK1 (PAK1), ER to nucleus signaling 1 (ERN1), and Glutathione Peroxidase 1(GPX1), which are related to oxidative stress and reactive oxygen species processes (n = 3 per group; mean ± SEM, adjusted p-value was calculated by DESeq2, Wald-test). (C) Western blotting analysis of NADPH oxidase pathway proteins expression in sh-NC and sh-SPARC HL60 cells. Quantification analysis of these proteins with GAPDH as an internal control. Relative expression was analyzed using ImageJ (version 1.53r) (n = 4 per group; mean ± SEM; Student's t-test: SPARC, protein kinase C (PKC), cytochrome B-245 alpha chain (CYBA), rac family small GTPase1+2+3 (Rac1+2+3), neutrophil cytosol factor 1 (NCF1), p-NCF1, p-NCF4, extracellular signal-regulated kinase (ERK), p-MEK; Welch's t-test: p-PKC, cytochrome b-245 beta chain (CYBB), neutrophil cytosol factor 2 (NCF2), neutrophil cytosol factor 4 (NCF4), p-ERK, mitogen-activated extracellular signal-regulated kinase (MEK)). (D) Detection of intracellular reactive oxygen species (ROS) levels in unstimulated sh-SPARC and sh-NC HL60 cells by flow cytometry. (E) Mean fluorescence intensity of intracellular ROS levels in unstimulated sh-SPARC and sh-NC HL60 cells (n = 4 per group; mean ± SEM, Welch's t-test). (F) After SPARC knockdown, the expression of NADPH oxidase pathway-related genes was detected by quantitative real-time polymerase chain reaction (RT-qPCR) (n = 4 per group; mean ± SEM, Student's t-test). Non-significant (ns) $p > 0.05$, * $p < 0.05$, ** $p < 0.01$, *** $p < 0.001$, **** $p < 0.0001$.

Supplementary Figure S6

A

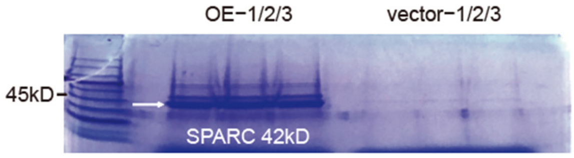

B

IP-MS/MS identified peptides of SPARC-interacting protein

	OE−1	OE−2	OE−3	vec−1	vec−2	vec−3
SPARC	21	25	21	3	3	4
RACK1	8	12	12	0	0	0

C

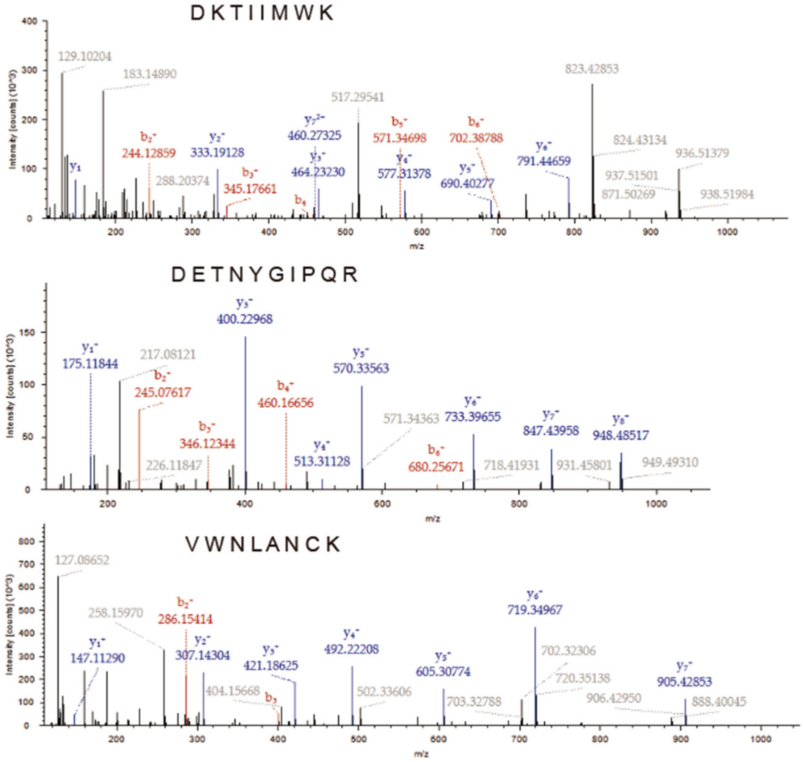

D

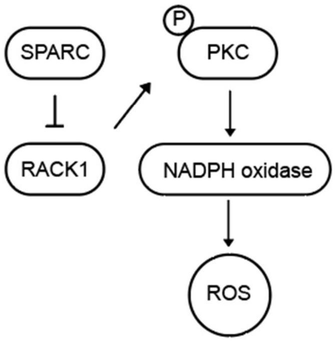

E

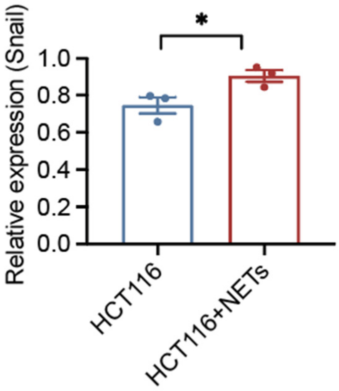

F

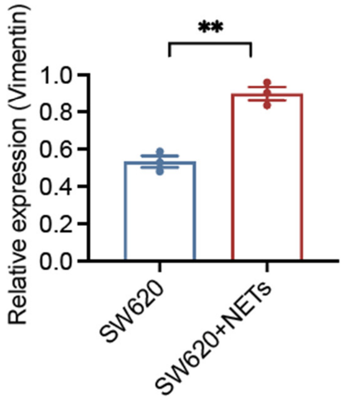

Supplementary Figure S6. Identification of SPARC interacting proteins. (A) Coomassie Brilliant Blue-stained SDS-PAGE gel for tandem mass spectrometry. (B) Immunoprecipitation-mass spectrometry identified SPARC-interacting proteins. (C) Representative peptide spectrums of RACK1. (D) Schematic diagram of the SPARC-regulated NADPH oxidase pathway. (E) Quantification analysis of Snail expression in HCT116 cells and NETs-treated HCT116 cells with β-actin as an internal control. Relative expression was analyzed using ImageJ (version 1.53r) (n = 3 per group; mean ± SEM; Student's t-test). (F) Quantification analysis of Vimentin expression in SW620 cells and NETs-treated SW620 cells with β-actin as an internal control. Relative expression was analyzed using ImageJ (version 1.53r) (n = 3 per group; mean ± SEM; Student's t-test). Non-significant (ns) $p > 0.05$, * $p < 0.05$, ** $p < 0.01$.

Supplementary Figure S7

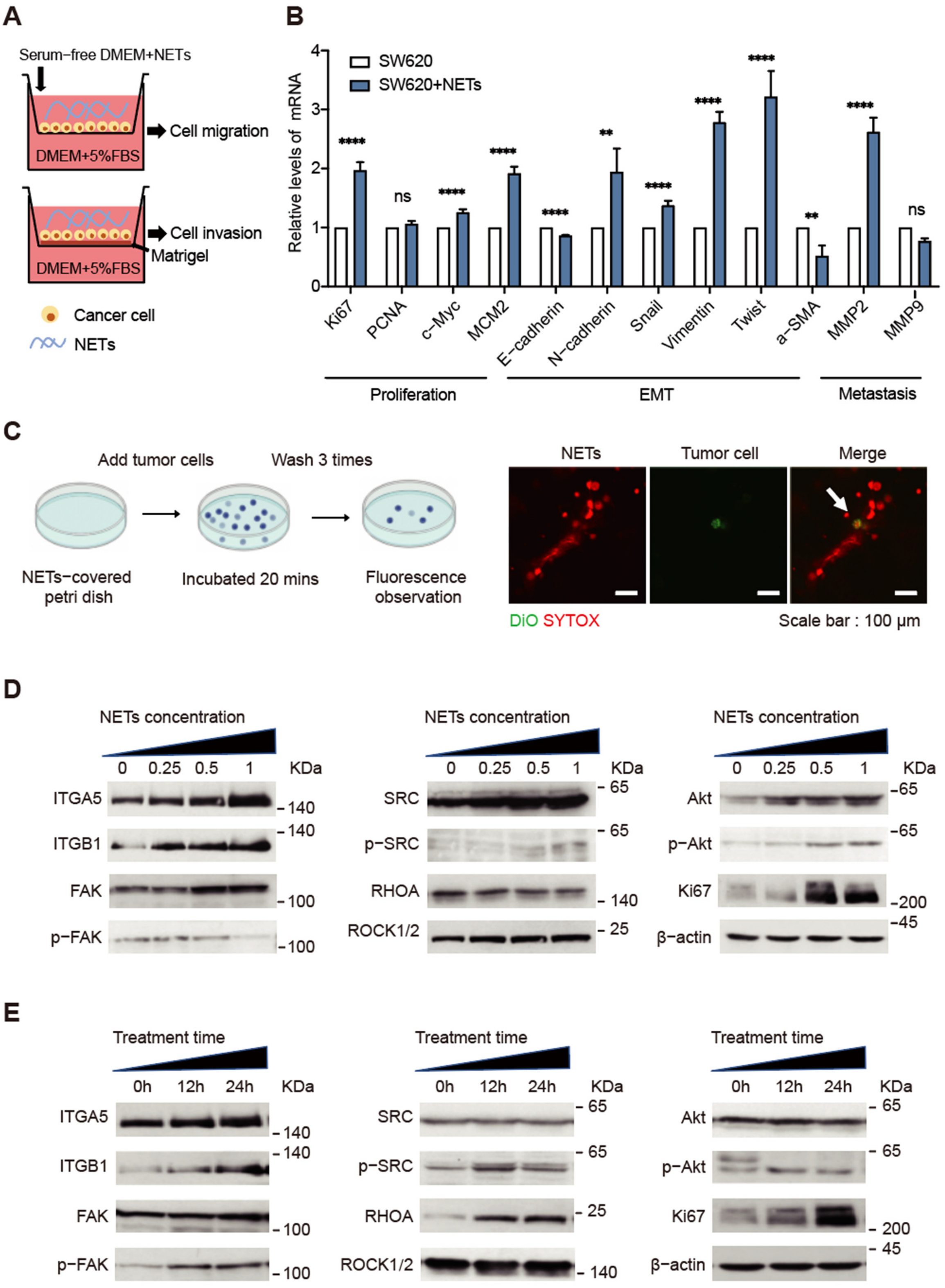

Supplementary Figure S7. Effects of NETs on CRC cells. (A) Schematic diagram of the transwell chamber assay. (B) RT-qPCR was used to detect genes related to proliferation and EMT after NETs incubation (n = 5 per group; mean ± SEM, Student's t-test). (C) Schematic diagram of the Static adhesion experiment (Left). Representative immunofluorescence images of NETs adhering to tumor cells *in vitro*. Red represents SYTOX Orange-labeled NETs and green represents DiO-labeled tumor cells (Right). Scale bar, 100 μm. (D) Western blotting analysis of integrin α5β1 signaling pathway proteins in HCT116 cells with NETs incubation. The degree of activation of the integrin α5β1 signaling pathway increases as the NETs concentration increases. (E) Western blotting analysis of integrin α5β1 signaling pathway proteins in HCT116 cells with NETs incubation. The degree of activation of the integrin signaling pathway increases as the NETs treatment time increases. Non-significant (ns) $p > 0.05$, ** $p < 0.01$, **** $p < 0.0001$.

Supplementary Figure S8

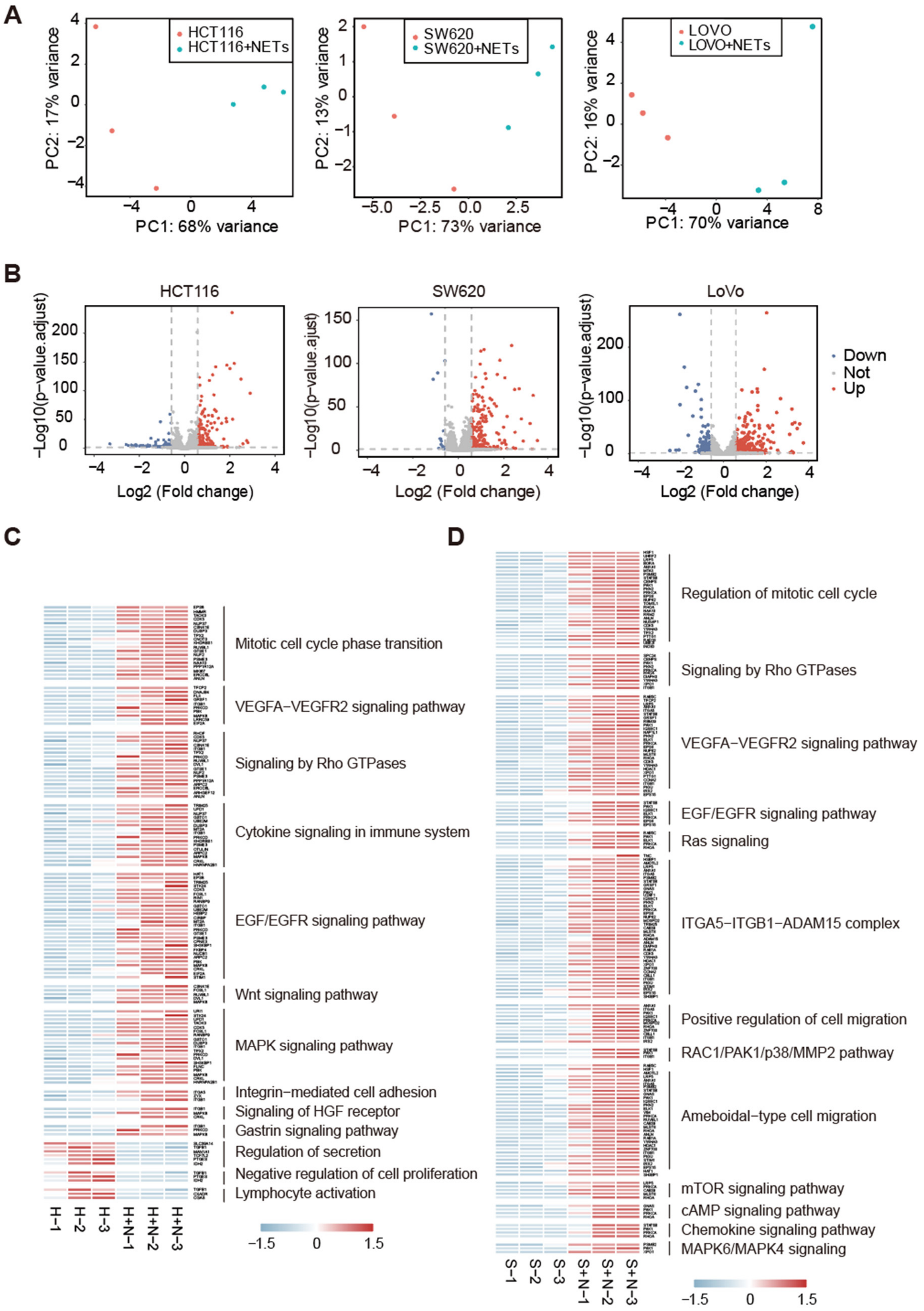

Supplementary Figure S8. Quantitative proteomics analysis of the effects of NETs on CRC cell lines. (A) Principal component analysis of proteomics data from different groups. (B) Volcano plots for the three CRC cell proteomes showing significantly changed proteins with an adjusted *p* value < 0.05 and an absolute value of Log2 (Fold change) > 0.5 (calculated as stated in the methods). Red represents significantly upregulated proteins and blue represents significantly downregulated proteins. (C) Biological function annotation enrichment analysis of significantly changed proteins in HCT116 cells. (D) Biological function annotation enrichment analysis of significantly changed proteins in SW620 cells.

Supplementary Figure S9

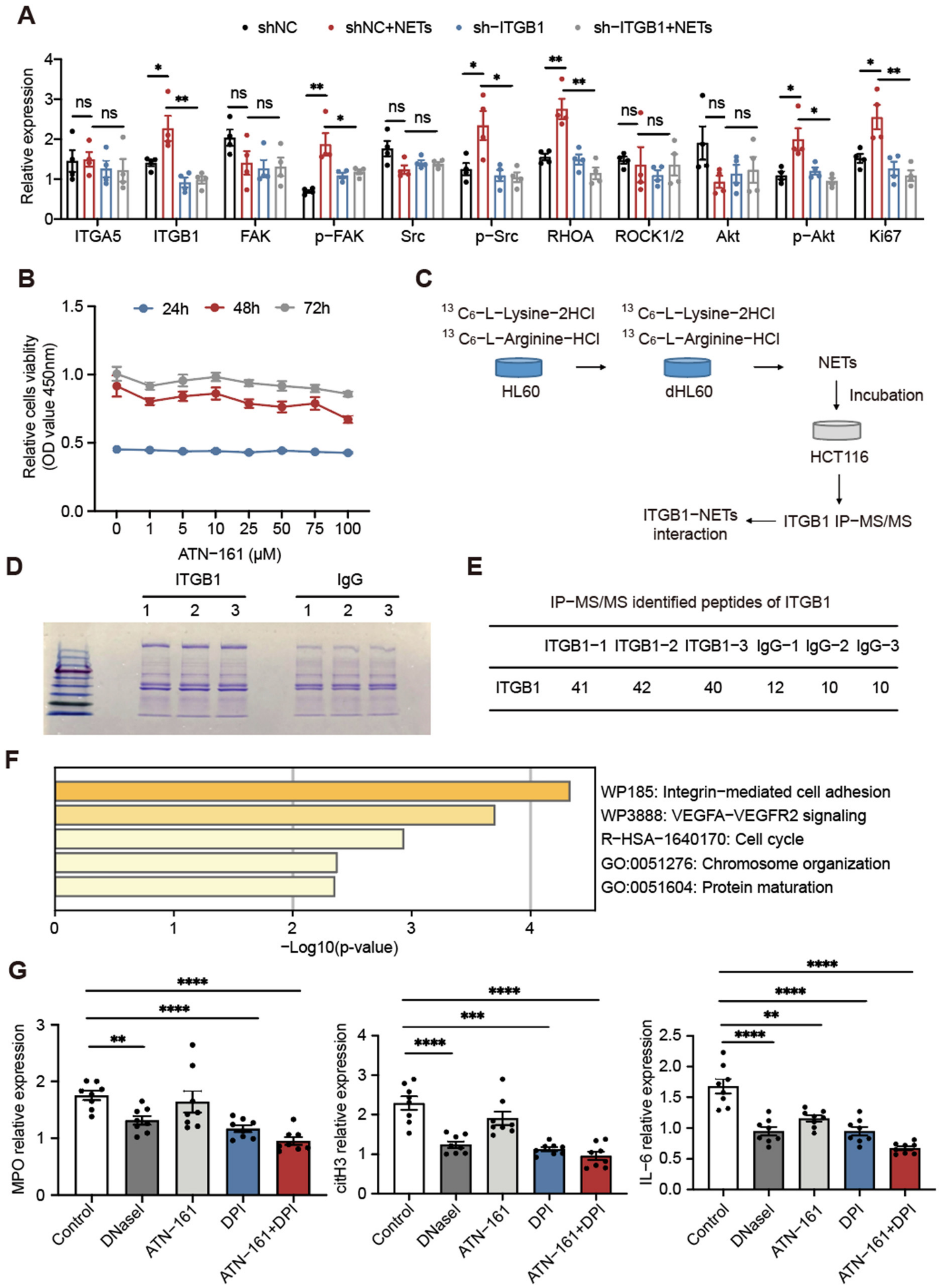

E

IP−MS/MS identified peptides of ITGB1

	ITGB1−1	ITGB1−2	ITGB1−3	IgG−1	IgG−2	IgG−3
ITGB1	41	42	40	12	10	10

Supplementary Figure S9. Proteins from NETs may interact with ITGB1 on tumor cells. (A) Quantification analysis of integrin α5β1 pathway proteins expression in sh-NC, sh-NC+NETs, sh-ITGB1, sh-ITGB1+NETs HCT116 cells with β-actin as an internal control. Relative expression was analyzed using ImageJ (version 1.53r) (n = 4 per group; mean ± SEM; Student's t-test). (B) cell counting kit 8 (CCK8) assay showed ATN-161(Ac-PHSCN-NH2) does not affect proliferation of HCT116 cells. (C) Experimental design for the identification of ITGB1-interacting proteins on NETs. (D) Coomassie brilliant blue-stained SDS-PAGE gel for tandem mass spectrometry. (E) Immunoprecipitation-mass spectrometry identified integrin beta-1 (ITGB1). (F) Biological function annotation enrichment analysis of NETs protein interact with ITGB1. (G) Quantification analysis of MPO, citH3 and IL-6 expression in plasma from different treatment groups of mice with ponceau S (Ponc.S) as an internal control. Relative expression was analyzed using ImageJ (version 1.53r) (n = 8 per group; mean ± SEM; Student's t-test). Non-significant (ns) $p > 0.05$, * $p < 0.05$, ** $p < 0.01$, *** $p < 0.001$, **** $p < 0.0001$.
